# Hypoxia Impairs Primordial Germ Cell Migration in Zebrafish (*Danio rerio*) Embryos

**DOI:** 10.1371/journal.pone.0024540

**Published:** 2011-09-08

**Authors:** Kwok Hong Lo, Michelle Nga Yu Hui, Richard Man Kit Yu, Rudolf Shiu Sun Wu, Shuk Han Cheng

**Affiliations:** 1 Department of Biology and Chemistry, City University of Hong Kong, Kowloon, Hong Kong SAR, China; 2 The State Key Lab in Marine Pollution, Department of Biology and Chemistry, City University of Hong Kong, Kowloon, Hong Kong SAR, China; 3 School of Environmental and Life Sciences, The University of Newcastle, Callaghan, Australia; Hong Kong University of Science and Technology, China

## Abstract

**Background:**

As a global environmental concern, hypoxia is known to be associated with many biological and physiological impairments in aquatic ecosystems. Previous studies have mainly focused on the effect of hypoxia in adult animals. However, the effect of hypoxia and the underlying mechanism of how hypoxia affects embryonic development of aquatic animals remain unclear.

**Methodology/Principal Findings:**

In the current study, the effect of hypoxia on primordial germ cell (PGC) migration in zebrafish embryos was investigated. Hypoxic embryos showed PGC migration defect as indicated by the presence of mis-migrated ectopic PGCs. Insulin-like growth factor (IGF) signaling is required for embryonic germ line development. Using real-time PCR, we found that the mRNA expression levels of insulin-like growth factor binding protein (IGFBP-1), an inhibitor of IGF bioactivity, were significantly increased in hypoxic embryos. Morpholino knockdown of IGFBP-1 rescued the PGC migration defect phenotype in hypoxic embryos, suggesting the role of IGFBP-1 in inducing PGC mis-migration.

**Conclusions/Significance:**

This study provides novel evidence that hypoxia disrupts PGC migration during embryonic development in fish. IGF signaling is shown to be one of the possible mechanisms for the causal link between hypoxia and PGC migration. We propose that hypoxia causes PGC migration defect by inhibiting IGF signaling through the induction of IGFBP-1.

## Introduction

Oxygen is essential to all vertebrate and invertebrate life. Widespread occurrence of hypoxia is of increasing environmental concern, and is a major threat to coastal ecosystems globally. Hypoxia in environment can be the result of multiple factors but pollution and eutrophication are most concerned. Hypoxic stress causes different metabolic changes in aerobic organisms, in which metabolic suppression is crucial for organisms to adapt to hypoxia. Evidence has shown that the key to adaptation to chronic hypoxia is a simultaneous reduction in metabolic rate and metabolic demands, i.e., by reducing or suspending many bioenergetic processes [1], [2]. Thus, the importance of understanding the effects of hypoxia is not limited to environmental studies but is extended to cell biology, physiology and developmental biology.

In recent years, there has been increasing research on the effect of hypoxia on reproduction in adult fish. Chronic aquatic hypoxia is shown to act as an endocrine disruptor, which leads to various types of reproduction impairment [3]. Long-term hypoxia exposure dramatically decreases gonadal growth and gametogenesis in Atlantic croaker, leading ultimately to reduction in fertility [4]. In *Fundulus frandis*, reduced fertility and delayed spawning are observed in hypoxia-exposed fish as compared to control normoxic fish [5]. To date, considerable attention has been focused on the effect of hypoxia in adult fish. However, the effect of hypoxia and the underlying mechanism of how hypoxia affects embryonic development still remain unclear.

Hypoxic stress influences human embryonic development [6] and the pathogenesis of several human diseases [7]. Insulin-like growth factors (IGFs) are well known fetal growth factors [8], [9]. Recent studies suggest that hypoxia inhibits fetal growth through inhibiting IGF signaling. Insulin-like growth factor binding protein 1 (IGFBP-1) is a secreted protein that binds to and inhibits IGF-1 and IGF-2 with high affinity. There is evidence for a pathophysiological role for IGFBP-1 in hypoxia-induced intrauterine growth restriction (IUGR) through inhibiting the growth-promoting activities of fetal IGFs [6], [10]. In zebrafish embryos, IGFBP-1 gene expression is hypoxia-inducible (through the action of hypoxia-inducible factor 1 (HIF-1)) [11] and knockdown of IGFBP-1 alleviates hypoxia-induced growth retardation and developmental delay [12]. Furthermore, re-introduction of IGFBP-1 to the IGFBP-1 knockdown embryos is able to restore the hypoxic effects on embryonic growth and development [12].

IGF signaling is also required for embryonic germ line development. Knockdown of IGF receptor b (IGF1R-b) has resulted in defective migration and elimination of primordial germ cells (PGCs), resulting in fewer PGCs colonizing at the genital ridges [13]. Germ line represents cells that carry the genetic material to the next generation [14]. The presumptive primordial germ cells (pPGCs) are germ line precursors that divide as stem cells during early cleavages. This asymmetric cell division gives rise to two daughter cells, one of which maintains the germ line stem cell character and the other one enters somatic lineages. PGCs are the founders of the germ line upon division, giving rise to daughter cells that contribute to the germ cell lineage. Germ cells are gonadal cells that reside in the gonad and give rise to gametes. The study on germ line is mainly focused on PGC development due to its unique cell cycle program during proliferation and migration. In early development, PGCs segregate from the somatic cells following a distinct cell division pattern and migrate to the future gonad site. The unique features of PGCs make them an attractive system to study the cell fate specification, differentiation and migration.

In this study, we used zebrafish embryos to investigate how hypoxia affects embryonic germ line development. The zebrafish is an excellent model for *in vivo* studies of vertebrate germ line because of the availability of molecular markers and the transparency of developing zebrafish embryos, which permits direct visualization of PGC development, a coordinated sequence of events involving cell fate specification, proliferation, and directed migration. Moreover, a number of conserved signaling pathways required for PGC development have been identified in zebrafish [15]–[18]. Here, we have provided evidence that zebrafish embryos showed defect in PGC migration upon hypoxia exposure. Furthermore, we have identified the IGF signaling pathway as one of the molecular mechanisms by which hypoxia disrupts PGC migration.

## Results

### Hypoxia impairs PGC migration in zebrafish embryos

**Figure 1:**
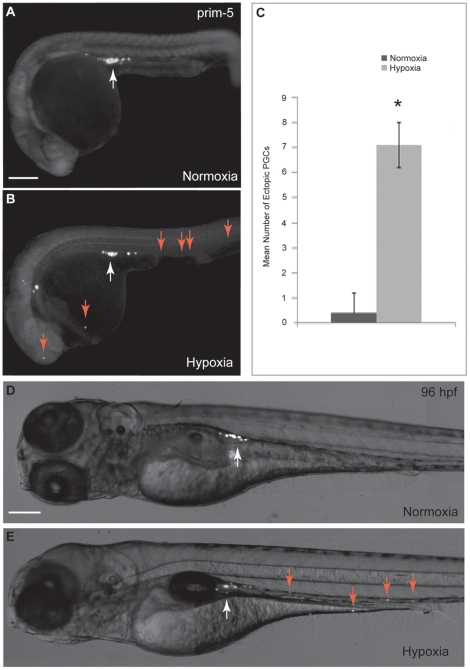
Hypoxia affects PGC migration in zebrafish embryos. Embryos were injected with GFP-*nos*l 3′UTR mRNA as PGC marker. (A) PGCs in normoxic embryos migrated properly towards the genital ridge at prim-5 stage (white arrow). (B) PGC migration is affected by hypoxia as illustrated by presence of mis-migrated ectopic PGCs at prim-5 stage (red arrows). (C) Significantly greater number of mis-migrated ectopic PGCs was observed in hypoxic embryos as compared with normoxic embryos at prim-5 stage. (D) PGCs in normoxic embryos located at the genital ridge at 96 hpf. (E) Mis-migrated ectopic PGCs in yolk sac and in cranial region remained in the same position and failed to move towards the genital ridge at 96 hpf (red arrows). Scale bar: 200 µm. * denotes *p*<0.05.

Embryos injected with GFP-*nos*l 3′UTR mRNA were exposed to either normoxia or hypoxia and were examined at prim-5 stage. The *nos*l 3′UTR sequence has been proven to be specifically stabilized in PGCs [19] and can be used as a tool to visualize PGCs *in vivo* in zebrafish embryos [20]. The results showed that all the PGCs in normoxic embryos were detected in the genital ridge at prim-5 stage (Figure 1A). However, under hypoxia, some PGCs were observed in cranial regions, dorsal caudal regions and yolk sac distal to the genital ridges (Figure 1B). This observation was similar to the previous description of mis-migrated ectopic PGCs [13], thus those PGCs not located in the genital ridge are regarded as mis-migrated ectopic PGCs hereafter. The number of mis-migrated ectopic PGCs in embryos exposed to normoxia or hypoxia was determined by visual counting under fluorescent microscope. At prim-5 stage, significantly more mis-migrated ectopic PGCs were observed in hypoxic embryos (7.1±0.9) than in normoxic embryos (0.4±0.8) (Figure 1C). After counting the number of mis-migrated ectopic PGCs in hypoxic embryos, those embryos with ectopic PGCs were re-exposed to hypoxia until 96 hours post fertilization (hpf). The mis-migrated ectopic PGCs in yolk sac and in cranial region remained in the same positions and failed to move towards the genital ridge (Figure 1E).

**Figure 2:**
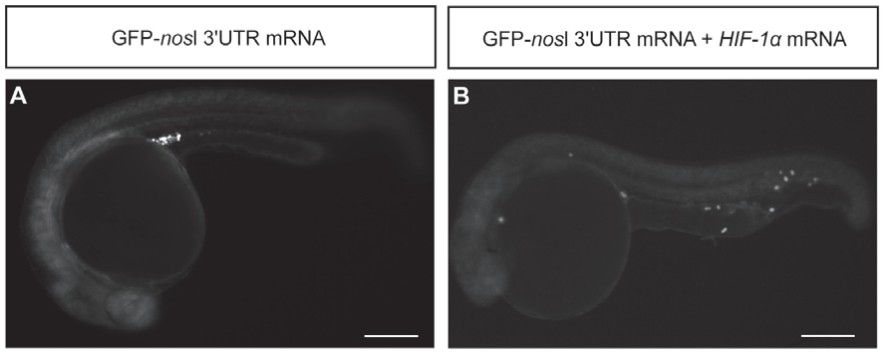
Effect of *HIF-1α* mRNA over-expression on PGC migration in zebrafish embryos. (A) Embryos injected with GFP-*nos*l 3′UTR mRNA. (B) Embryos co-injected with GFP-*nos*l 3′UTR mRNA and *HIF-1α* mRNA. *HIF-1α* over-expressed embryos showed mis-migrated ectopic PGCs in the head, yolk sac extension and caudal region. *HIF-1α* over-expressed embryos displayed similar phenotypes as embryos exposed to hypoxia. Scale bar: 200 µm.

To observe the behavior of PGCs during their migration to the genital ridge in zebrafish embryos exposed to either normoxia or hypoxia, we live monitored the PGC migration process under fluorescent microscope. Images were captured every 5 min from the beginning of gastrulation, which involves the formation of germ ring and subsequently the embryonic shield, to prim-5 stage. Then, the time-lapse images were transformed into videos. Embryos were injected with GFP-*nos*l 3′UTR mRNA, which enabled the live monitoring of PGC migration in zebrafish embryos. The hypoxic condition was produced by injection of *HIF-1α* mRNA in order to overcome the technical problem of live monitoring embryos under hypoxia. HIF-1α is a master regulator of hypoxia signaling and is stabilized under hypoxia [21]. The phenotype resulted from *HIF-1α* mRNA over-expression was similar to the phenotype of hypoxia-induced mis-migrated ectopic PGCs. At prim-5 stage, mis-migrated ectopic PGCs were detected in the cranial region, yolk sac extension and caudal region in embryos co-injected with GFP-*nos*l mRNA and *HIF-1α* mRNA as compared with embryos injected with GFP-*nos*l 3′UTR mRNA alone (Figure 2). Hence, these results suggest that PGC migration defect upon hypoxia exposure is mediated by HIF-1α.

As shown in the time-lapse video (Video S1), under normoxia, four clusters of PGC were found in embryos at 70% epiboly. From the lateral view, two clusters of PGCs were located on the dorsal side and the ventral side. The two clusters of PGCs moved towards the intermediate target within the lateral mesoderm and joined together at bud stage. Finally, they aligned themselves within the lateral mesoderm. In *HIF-1α* mRNA-injected embryos (Video S2), PGCs were randomly distributed at 90% epiboly and then moved towards the dorsal side of the embryo, aligning at a dorsal-lateral position. PGCs near the first forming somite migrated towards the forming notochord and aligned themselves there with proliferation. However, PGCs distant from the forming notochord failed to move towards it, which explains the origin of ectopic PGCs in hypoxic embryos.

### Hypoxia does not promote cell death in PGCs

**Figure 3:**
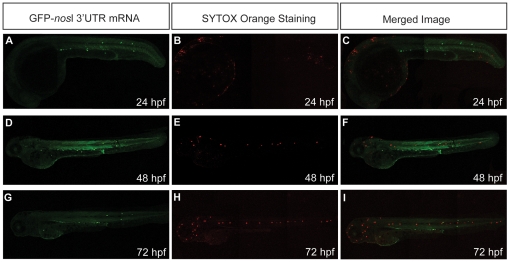
Hypoxia does not promote cell death in mis-migrated ectopic PGCs. (A–C) Embryos injected with GFP-*nos*l 3′UTR mRNA were exposed to hypoxia and stained with SYTOX orange for dead cells. Mis-migrated ectopic PGCs (green) were not removed by apoptosis. The merged images show no co-localization between mis-migrated ectopic PGCs (green) and dead cells stained by STYOX Orange (red).

Embryos injected with GFP-*nos*l 3′UTR mRNA as a PGC marker were exposed to hypoxia until prim-5 stage. Hypoxic embryos were then incubated with SYTOX Orange to stain for the nucleic acids of dead cells to investigate whether the mis-migrated ectopic PGCs were removed by cell death. Our results demonstrated that neither mis-migrated ectopic PGCs nor normal PGCs in the genital ridge in hypoxic embryos were positively stained by SYTOX Orange at prim-5 stage (24 hpf), 48 and 72 hpf (Figure 3). This indicated that under hypoxia, the mis-migrated ectopic PGCs in zebrafish embryos were not removed by cell death.

### IGFBP-1 is hypoxia-inducible and mediates PGC mis-migration in hypoxic zebrafish embryos

**Figure 4:**
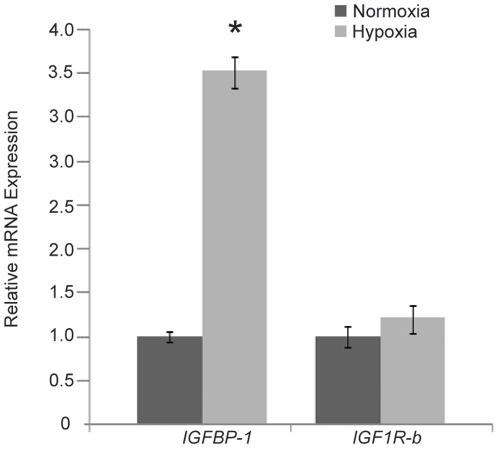
Hypoxia induces IGFBP-1 mRNA expression in zebrafish embryos at prim-5 stage. Expression levels of *IGFBP-1* mRNA and *IGF1R-b* mRNA were quantified using real-time PCR and normalized against 18S rRNA. Data are the mean relative fold changes ± SE (n = 10) with respect to the control (normoxia) level (arbitrarily set to 1). Significantly higher *IGFBP-1* mRNA expression was found in hypoxic embryos as compared with normoxic embryos. No significant difference in *IGF1R-b* mRNA expression was found between normoxic and hypoxic embryos. * denotes *p*<0.05.

**Figure 5:**
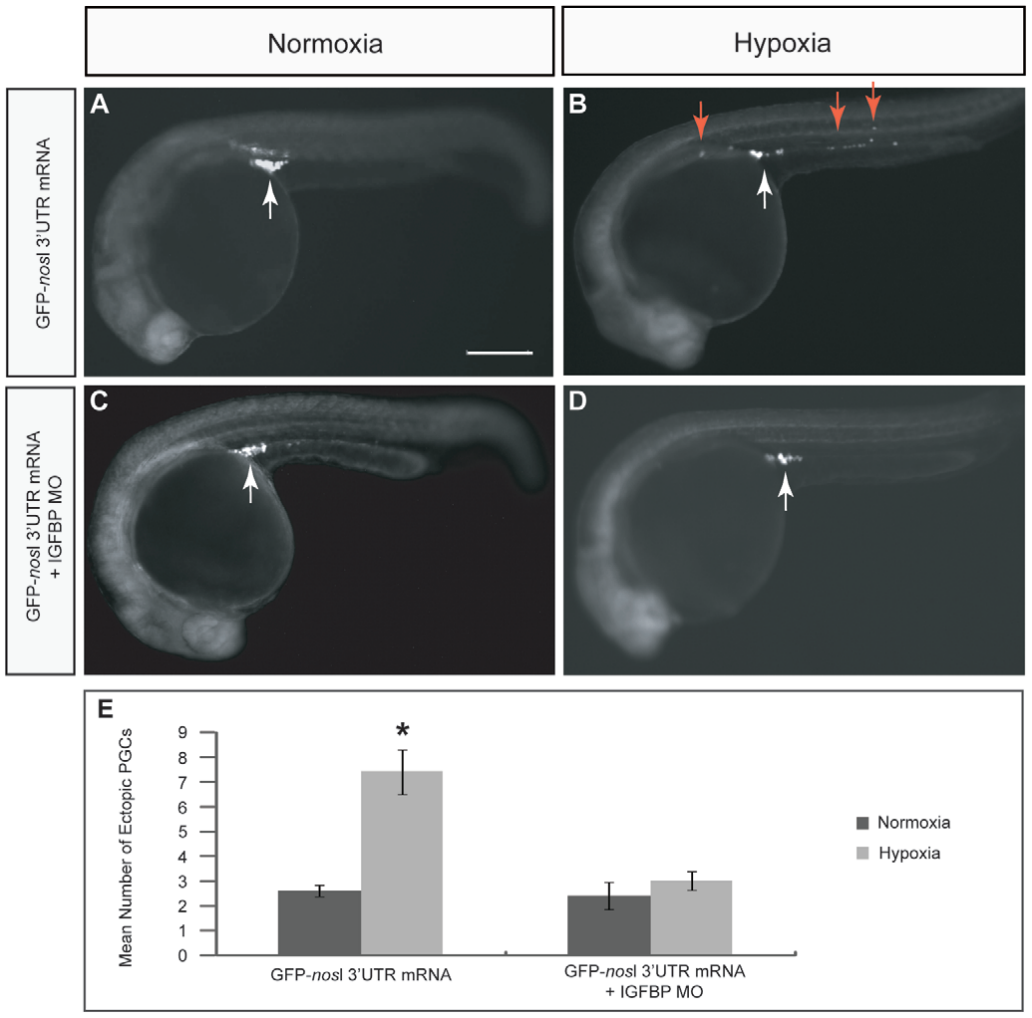
Effect of IGFBP-1 knockdown on PGC migration in zebrafish embryos. (A and B) Embryos were injected with GFP-*nos*l 3′UTR mRNA alone and subsequently exposed to either normoxia or hypoxia. PGC migration was impaired in hypoxic embryos (B) as compared with normoxic embryos (A). (C and D) Embryos were co-injected with GFP-*nos*l 3′UTR mRNA and IGFBP-1 MO and subsequently exposed to either normoxia or hypoxia. (D) PGC migration defect in hypoxic embryos was minimized by IGFBP-1 MO knockdown. Scale bar: 200 µm. (E) Mean number of mis-migrated ectopic PGCs in normoxia and hypoxia embryos injected with GFP-*nos*l 3′UTR mRNA alone or co-injected with GFP-*nos*l 3′UTR mRNA and IGFBP-1 MO. The mis-migrated ectopic PGCs phenotype could be rescued by IGFBP-1 MO knockdown as indicated by reduction in number of mis-migrated ectopic PGCs in hypoxic embryos. * denotes *p*<0.05.

IGF signaling is known to be important for PGC migration and survival in zebrafish embryos. Knockdown of *IGF1R-b* results in ectopic PGCs and reduced number of PGCs in the genital ridge in zebrafish [13]. Since HIF-1 is able to transactivate IGFBP-1 gene expression [11], we hypothesized that hypoxia induces IGFBP-1, which in turn suppresses the IGF signaling and results in PGC mis-migration. To test this hypothesis, real-time PCR was used to quantify the mRNA expression levels of *IGFBP-1* and *IGF1R-b* in zebrafish embryos at prim-5 stage after exposure to normoxia or hypoxia (Figure 4). *IGFBP-1* was significantly up-regulated in hypoxic-exposed embryos. However, no significant difference was found in the *IGF1R-b* expression between normoxic and hypoxic embryos. To test whether IGFBP-1 impairs PGC development under hypoxia, embryos were injected with GFP-*nos*l 3′UTR mRNA alone or co-injected with GFP-*nos*l 3′UTR mRNA with IGFBP-1 morpholino (MO), followed by normoxia or hypoxia exposure and examined at prim-5 stage. No PGC migration defect was observed in embryos co-injected with GFP-*nos*l 3′UTR mRNA and IGFBP-1 MO and reared under normoxia (Figure 5C) or hypoxia (Figure 5D). The mean number of mis-migrated ectopic PGCs in IGFBP-1 morphants under hypoxia (3±0.4), control embryos under normoxia (2.6±0.2) and IGFBP-1 morphants under normoxia (2.4±0.6) were not significantly different from each other (Figure 5E). These data indicated that knockdown of IGFBP-1 abolishes the formation of ectopic PGCs in hypoxic embryos but has no effect on PGC migration in normoxic embryos. Taken together, our results suggest that hypoxia mediates PGC mis-migration through IGFBP-1.

## Discussion

This study provides the first evidence that hypoxia causes PGC migration defect in fish embryos. Significantly more mis-migrated ectopic PGCs were detected in hypoxic embryos in comparison with normoxic embryos. Along different developmental stages of hypoxic embryos, mis-migrated ectopic PGCs were detected in dorsal caudal regions and yolk cell distal to the genital ridges. This observation suggested that hypoxia induces occurrence of mis-migrated ectopic PGCs, an indication of PGC migration failure. In addition, we established that PGC mis-migration is related to the inhibition of IGF signaling due to the induction of IGFBP-1 by hypoxia.

### Mis-migrated ectopic PGCs

The time-lapse videos demonstrated that mis-migrated ectopic PGCs could be observed between 80% epiboly and 1-somite stage. It is generally believed that chemotaxis is involved in PGC migration. Chemotaxis is the ability of cells to sense and migrate along an external chemical gradient. This process requires the integration of different cell responses including motility, polarization and directional sensing [22], [23]. Suppression of IGF signaling leads to quantitative changes in the expression of genes encoding CXCL-family chemokine ligands and receptors involved in PGC migration [13]. In zebrafish, PGC migration relies on directional cues provided by somatic tissues on their way to the gonad [24]–[26]. Previous work demonstrated that directional PGC migration requires the chemokine SDF-1 [16] and its receptor Cxcr4b [27]. The attractant SDF-1 and its receptor CXCR4b comprise a system for guiding vertebrate PGCs throughout the PGC migration process [27]. In zebrafish, both the *odysseus* (*ody*) mutant, having the complete loss of CXCR4b protein function, and knockdown of SDF-1 showed PGC migration defect as illustrated by increased ectopic PGCs [27]. The hypoxia-induced expression of CXCR4 relies on both transcriptional and posttranscriptional mechanisms in which HIF-1α is found to interact with CXCR4 promoter and CXCR4 mRNA stability is increased by hypoxia, respectively [28].

PGC migration in normal circumstances can be summarized into four steps. First, PGCs move dorsally during gastrulation and then form a line at the border between the head and trunk mesoderm. Then, PGCs move towards an intermediate target within the lateral plate mesoderm. Finally, PGCs leave this intermediate target and migrate posteriorly to colonize in the gonad. SDF-1 is expressed in a stripe between the head and trunk mesoderm at the time PGCs accumulate at this position. Later, SDF-1 expression becomes elevated in the lateral mesoderm surrounding the intermediate target. Finally, its expression recedes posteriorly as PGCs migrate toward the gonad. Morpholino knockdown of either the receptor or the ligand abrogates directional PGC migration and leads to ectopic localization of these cells [16]. Besides, ectopic expression of SDF-1 could lure germ cells away from their normal migration routes.

Although the critical time period leading to the formation of ectopic PGCs was determined, it would be interesting to know how hypoxia leads to PGC migration defect during gastrulation. There are a few developmental defects that could lead to PGC migratory defects. One or more of these could explain the migration defect phenotype observed in hypoxic embryos: (1) An intrinsic loss of cell motility, leaving PGCs unable to respond to extrinsic migratory cues; (2) an intrinsic failure to correctly interpret extrinsic cues; (3) defective development or death of somatic cells that provide guidance cues to migratory PGCs; and (4) improper cell-cell adhesion between PGCs and somatic cells. The time-lapse videos showed that mis-migrated ectopic PGCs were able to move, thus PGC migration defect could not be a consequence of cell immotility. Regulation of CXCR4 expression by hypoxia may be essential for directing PGC migration. It has been reported that hypoxic preconditioning induces CXCR4 expression in mesenchymal stell cells [29]. However, our video shows that only PGCs that are near the posterior side cannot migrate towards the intermediate target during gastrulation. It is unclear why only those PGCs cannot migrate correctly. Therefore, the second possibility could also be ruled out.

The third possibility is that under hypoxia defective development or death of somatic cells provides guidance cues to migratory PGCs. As reported, the major biological effects of the chemokine SDF-1a are related to its ability to induce motility, chemotactic responses and adhesion in cells bearing cognate CXCR4 [30]. Cells bearing CXCR4 always respond to a SDF-1 gradient produced by somatic cells [16]. If hypoxia disrupts the SDF-1 gradient produced by somatic cells in the intermediate target, it is possible that only the PGC that are close to the intermediate target would respond to this cue, while PGCs distant from the intermediate target would fail to do so. Future studies are required to investigate this possibility.

The fourth possibility, improper cell-cell adhesion between PGCs and somatic cells, could also explain PGC migration defect during gastrulation. It is suggested that the PGC movement to the intermediate target during gastrulation depends on general gastrulation movement. The segregation of individual cells from the tissues where they originally reside requires alterations in their adhesive properties. Modulations of cell-cell interactions leading to cell detachment and invasion of neighboring tissues has been shown to promote dispersion of tumor cells and to be essential for morphogenesis during normal development [31]. A molecule known to play a critical role in controlling cell-cell adhesion in such biological contexts is the calcium-dependent cell adhesion molecule, E-cadherin [32]. It has been shown that the level of membranal E-cadherin is modulated during early PGC development and reduction in E-cadherin is important for PGC motility. E-cadherin is down-regulated on the membrane of PGCs upon the onset of migration and its expression persists in the cells as they migrate. However, a high level of E-cadherin makes PGC immotile [33]. It has been reported that E-cadherin down-regulation is induced by hypoxia in trophoblast cells [34] and in ovarian carcinoma cells [35]. Therefore, it is possible that hypoxia alters PGC membranal E-cadherin level.

### IGFBP-1 is involved in PGC migration defect in hypoxic embryos

In this study, we also established that hypoxia-induced PGC mis-migration is related to the inhibition of IGF signaling due to the induction of IGFBP-1 by hypoxia. Both IGF1R-b and IGFBP-1 play an important role in IGF signaling, which is essential for PGC migration. The real-time PCR results showed no significant difference in *IGF1R-b* expression between hypoxic embryos and normoxic embryos, while *IGFBP-1* expression was up-regulated in hypoxic embryos. Therefore, it is possible that the induced IGFBP-1 expression is involved in PGC migration defect observed in hypoxic embryos. If an increased IGFBP-1 level is responsible for PGC migration defect in hypoxic embryos, IGFBP-1 knockdown should rescue the PGC migration defect. There was no significant difference in the mean number of mis-migrated ectopic PGCs between hypoxic embryos co-injected with IGFBP-1 MO and GFP-*nosl* 3′UTR mRNA and normoxic embryos injected only with GFP-*nosl* 3′UTR mRNA, demonstrating that hypoxia-induced PGC migration defect could be rescued by knockdown of IGFBP-1. Although the IGFBP-1 transcript can be detected throughout embryogenesis, no strong IGFBP-1 mRNA signal can be detected in embryos of cleavage, blastula and gastrula stages [36]. Furthermore, hypoxia has been shown to stimulate ubiquitous expression of IGFBP-1 at 24 and 48 hpf [12]. Despite that hypoxia has been shown to induce IGFBP-1 expression by quantitative PCR, no specific expression pattern of IGFBP-1 would be observable during the PGC migration process which occurs during gastrulation stage to prim-5 stage. Taken together, our findings suggest that increased levels of IGFBP-1 induced by hypoxia lead to inhibition of IGF signaling, and this inhibition in turn results in PGC migration in zebrafish embryos. PGC migration in zebrafish embryos is sensitive to local aberrations in IGF signaling [37]. PGC-specific over-expression of any one of the three IGF ligands (IGF-I, IGF-IIa, IGF-IIb) shows no effect on the total PGC number, but potently disrupts their migration, leading to greater numbers of mis-migrated PGCs [36]. PGCs-specific suppression of IGF signaling had no detectable effects on somatic tissues, but deleterious effects on directional PGC migration [37]. These findings support the notion that PGCs intrinsically require IGF signaling to migrate correctly. In addition to its significance in the PGC microenvironment, IGF signaling is also required for development of somatic tissues in the urogenital tract, particularly considering the strong expression of *igf2b* in the nephron primordia and the presumptive pronephric ducts. The importance of somatic tissue development for PGC migration is revealed by analyses of zebrafish mutants with specific defects in mesoderm development [38], [39]. Failure of PGCs to aggregate at their intermediate clustering site (pronephric mesoderm) in *one-eyed-pinhead*;*notail* (*oep*;*ntl*) and *spadetail*;*notail* (*spt*;*ntl*) double mutants is a defect attributed to improper differentiation of pronephric tissues. Similarly, PGCs in *hands off* mutants (*han*), which exhibit defects in gonadal mesoderm development, fail to migrate from the intermediate target site to the embryonic gonad. In each of these cases, aberrant PGC migration could ultimately be attributed to a failure in the development of somatic tissues, which require chemotactic signals to emanate.

### Hypoxia does not promote cell death of mis-migrated ectopic PGCs

Studies have indicated that ectopic PGCs are selectively removed by apoptosis [13], [19]. However, mis-migrated ectopic PGCs in the yolk cell and in the cranial region remained in the same positions in hypoxic embryos at 96 hpf (Figure 1D). No positive staining of dead cells by Sytox Orange was found to co-localize with mis-migrated ectopic PGCs nor normal PGCs, indicating that hypoxia does not promote cell death of normal PGCs nor ectopic PGCs. The absence of cell death may be explained by the fact that apoptosis is an energy demanding process and requires a series of energy demanding activities such as p53 accumulation, followed by Bcl-xL down-regulation, cytochrome c release and caspase activation [40]. Studies showed that hypoxia suppresses apoptosis by inducing nucleophosmin (NPM), which suppresses p53 and hence prevents apoptosis [41], [42].

### Implication of PGC migration defect caused by hypoxia

Although fertility defects may be the secondary manifestation of systemic illnesses, often the defect is primary to the reproductive system [43]. PGCs are cells that differentiate into germ cells and become gametes after gametogenesis. The presence of PGCs is important for differentiation and survival of the gonad [44]. PGC migration and colonization in the gonads are important developmental processes in which failure of PGCs to migrate correctly can lead to impaired gonadal development and to the formation of germ cell tumors [45], [46]. It is possible that hypoxia impairs PGC migration, leading to more ectopic PGCs and, in turn, reduces the PGC population in the genital ridge. Knockdown of *dead end*, a gene encoding putative RNA binding protein, is a component of the germ plasm, resulted in ablation of PGCs and the morphants all developed into sterile male adults [47]. This indicates that the number of PGCs in the gonad may be related to sex differentiation. A recent study also showed that hypoxia affects sex differentiation and development in zebrafish, leading to a male-dominated population [48]. Thus, PGC mis-migration in zebrafish embryos in response to hypoxia exposure may be a possible way leading to male-dominated population in fish.

## Materials and Methods

### Ethnics Statement

All embryos were handled in accordance with the licence for the control of experiments on animals approved by the Department of Health of the Government of the Hong Kong SAR (Ref (11-8) in DH/HA&P/8/2/5 Pt.1).

### Zebrafish Maintenance and Hypoxia Exposure

Adult zebrafish (Danio *rerio*) were maintained as previously described [49]. Fertilized eggs were collected at the first hour of light period in 14-h light/10-h dark cycle. Collected embryos were exposed to either normoxia or hypoxia for subsequent experiments. Normoxic and hypoxic conditions were maintained at dissolved oxygen (DO) level of 7.0±0.2 mg O_2_/L and 1.0±0.2 mg O_2_/L, respectively. The DO levels were monitored continuously using dissolved oxygen meters and polarographic probes (Cole-Parmer 5643-00, Illinois, USA). Upon exposure, normoxic and hypoxic embryos were examined and compared at the same developmental stages [50].

### Microinjection of mRNA and Morpholino Oligonucleotides

Capped and poly(A)-tailed mRNA was synthesized using an mMESSAGE mMACHINE kit (Ambion). DNA plasmid containing GFP *nos*1 3′UTR as PGCs marker was generously provided by Dr. Erez Raz [19]. The mRNA was diluted to 0.4 µg/µl with RNAase free water and injected in a volume of 2 nl into one-cell stage zebrafish embryos. Antisense morpholino oligonucleotides (MOs) were synthesized by Gene Tools, LLC (Corvallis, OR). Two splice-site-targeted IGFBP-1 MOs were designed: MO1 targeted on the sequence between the first exon and first intron of the coding region and MO2 targeted on the sequence between the third exon and third intron of the coding region. For the co-injection experiments, embryos were injected with capped mRNA and MO at optimized dosage.

### Time lapse imaging

Embryos injected with GFP *nos*1 3′UTR mRNA alone and embryos co-injected with GFP *nos*1 3′UTR mRNA and *HIF-1α* mRNA were live monitored under spinning disk confocal microscope (Olympus BX61 DSU) connected to EM-CCD camera. Images were captured every 5 min and transformed into video with Image-Pro Plus (MediaCybernetics, Inc.).

### SYTOX orange staining

Live embryos at desired developmental stages were incubated in 0.1 µM SYTOX Orange Nucleic Acid Stain (Molecular Probes) for 10 min. Embryos were then rinsed with PBS and examined with a fluorescent microscope.

### RNA Extraction and Reverse Transcription

Total RNA from embryos exposed to normoxia or hypoxia was extracted by TRIZOL (Invitrogen) according to the manufacturer's instructions. First-strand cDNA was synthesized using 1 µg total RNA, 1 µL random primers (250 ng), 1 µL dNTP (10 mM), 4 µL first-strand buffer, 2 µL 0.1 M DTT, 1 L RNaseOUT (40 U; Invitrogen, Carlsbad, CA), 1 µL SuperScript™ II Reverse Transcriptase (200 U; Invitrogen) in a total volume of 20 µL at 42°C for 50 min. The reaction was inactivated by heating at 70°C for 15 min. First-strand cDNA was diluted with water (1∶5) was used as a template for real-time PCR.

### Real-Time Polymerase Chain Reaction

Real-time PCR was performed according to Yu at el., 2008 [51]. Real-time PCR was performed using the iCycler iQ Real-time PCR System (BioRad) with SYBR Green I dye-based detection method. To normalize target gene expression for differences in cDNA input, cDNA was diluted 1∶5000 for measuring 18S rRNA levels. Diluted cDNAs (5 µL aliquots) were added as template in triplicate to the wells of a 96-well thin-wall PCR plate. To each well, 20 µL PCR master mix containing 12.5 µL 2× iQ SYBR Green Supermix (BioRad), 1 µL of each target gene primer or 18S rRNA primer (10 µM) and 5.5 µL water. Specific PCR primer sequences and reaction conditions are presented in Table S1. The PCR plate was heated at 95°C for 2 min followed by 40 cycles of 95°C for 20 s, 60°C for 30 s and 72°C for 30 s. For quantification of PCR results, C_T_ (the cycle at which the fluorescence signal is significantly different from background) was determined for each reaction.

### Statistical Analysis

Data were expressed as mean ± SEM. Statistical analyses were performed using SPSS (SPSS Inc., Chicago, IL, USA). Student *t*-test was used to evaluate the significance difference between normoxic and hypoxic groups. The level accepted for statistical significance in all cases was *p*<0.05.

## Supporting Information

Table S1 **Primers used for real-time PCR.** The sequences of forward and reverse primers and the product size for each gene tested in this study are included.
(TIF)

Video S1 **PGC migration in control zebrafish embryos by time-lapse imaging.** Control embryo was injected with GFP-*nos*l 3′UTR mRNA.
(WMV)

Video S2 **PGC migration in hypoxic zebrafish embryos by time-lapse imaging.** Hypoxic-stressed embryo was co-injected with GFP-*nos*l 3′UTR mRNA and *HIF-1α* mRNA.
(WMV)
