# Supplementary figures and images for: Hypoxia Impairs Primordial Germ Cell Migration in Zebrafish (Danio rerio) Embryos
Source: PLoS One. 2011 Sep 8;6(9):e24540. doi: 10.1371/journal.pone.0024540 (PMC3169607; doi:10.1371/journal.pone.0024540)

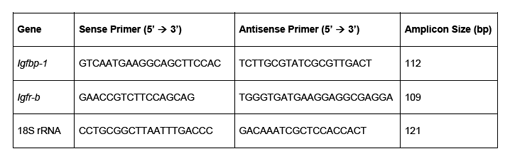

Supplement: Table S1 — Primers used for real-time PCR. The sequences of forward and reverse primers and the product size for each gene tested in this study are included. (TIF) [file pone.0024540.s001.tif]
